# Supplementary material for: The Effect of Strict Lockdown on Omicron SARS-CoV-2 Variant Transmission in Shanghai
Source: Vaccines (Basel). 2022 Aug 25;10(9):1392. doi: 10.3390/vaccines10091392 (PMC9500677; doi:10.3390/vaccines10091392)

Supplementary information for

The effect of strict lockdown on Omicron SARS-CoV-2 variant transmission in Shanghai

Supplementary Table S1. Calculating sub-index scores for indicators of metrics.

Name	Coding	Score
**C1(School closing)**	0 - no measures	0
	1 - recommend closing or all schools open with alterations resulting in significant differences compared to non-Covid-19 operations	33.33
	2 - require closing (only some levels or categories, eg just high school, or just public schools)	66.67
	3 - require closing all levels	100
**C2(Workplace closing)**	0 - no measures	0
	1 - recommend closing (or recommend work from home) or all businesses open with alterations resulting in significant differences compared to non-Covid-19 operation	33.33
	2 - require closing (or work from home) for some sectors or categories of workers	66.67
	3 - require closing (or work from home) for all-but-essential workplaces (eg grocery stores, doctors)	100
**C3(Cancel public events)**	0 - no measures	0
	1 - recommend cancelling	50
	2 - require cancelling	100
**C4(Restrictions on gatherings)**	0 - no restrictions	0
	1 - restrictions on very large gatherings (the limit is above 1000 people)	25
	2 - restrictions on gatherings between 101-1000 people	50
	3 - restrictions on gatherings between 11-100 people	75
	4 - restrictions on gatherings of 10 people or less	100
**C5(Close public transport)**	0 - no measures	0
	1 - recommend closing (or significantly reduce volume/route/means of transport available)	50
	2 - require closing (or prohibit most citizens from using it)	100
**C6(Stay at home requirements)**	0 - no measures	0
	1 - recommend not leaving house	33.33
	2 - require not leaving house with exceptions for daily exercise, grocery shopping, and 'essential' trips	66.67
	3 - require not leaving house with minimal exceptions (eg allowed to leave once a week, or only one person can leave at a time, etc)	100
**C7(Restrictions on internal movement)**	0 - no measures	0
	1 - recommend not to travel between regions/cities	50
	2 - internal movement restrictions in place	100
**C8(International travel controls)**	0 - no restrictions	0
	1 - screening arrivals	25
	2 - quarantine arrivals from some or all regions	50
	3 - ban arrivals from some regions	75

	4 - ban on all regions or total border closure	100

The scores of each indicator are calculated by the equation described in the website link (https://github.com/OxCGRT/covid-policy-tracker/blob/master/documentation/index_methodology.md).

Supplementary Table S2. Stringency indices of lockdown policies implemented by different countries.

Country	C1	C2	C3	C4	C5	C6	C7	C8	Stringency indices
**India**	100	100	100	100	100	100	100	100	100
**Italy**	100	100	100	100	100	100	100	75	96.88
**Shanghai, China**	100	100	100	100	100	100	100	75	96.88
**Germany**	100	100	100	100	50	100	100	75	90.62
**France**	100	100	100	100	50	100	100	75	90.62
**United States**	100	100	100	100	50	100	100	75	90.62
**Australia**	100	66.67	100	100	50	100	100	100	89.58
**Canada**	100	100	100	100	0	100	100	100	87.5
**Spain**	100	100	100	100	50	100	50	100	87.5
**Netherland**	100	100	100	100	50	100	50	75	84.37
**United Kingdom**	100	100	100	100	50	100	100	0	81.25
**Singapore**	100	100	100	100	0	66.67	100	75	80.21
**South Korea**	100	100	100	100	0	66.67	100	75	80.21
**Norway**	100	66.67	100	100	50	0	100	100	77.08
**Finland**	66.67	66.67	100	100	0	33.33	100	100	70.83
**Denmark**	100	66.67	50	100	50	33.33	50	100	68.75
**Sweden**	66.67	66.67	100	100	50	33.33	50	75	67.71
**Japan**	66.67	66.67	100	50	0	33.33	50	100	58.33

Stringency indices are simple averages of the individual component indicators.

Supplementary Table S3. Daily new infections in Shanghai from March 1st to April 30th, 2022.

Date	Daily symptomatic cases	Daily asymptomatic cases	Daily new infections
**Mar 01, 2022**	1	18	19
**Mar 02, 2022**	3	24	27
**Mar 03, 2022**	2	35	37
**Mar 04, 2022**	3	26	29
**Mar 05, 2022**	0	38	38
**Mar 06, 2022**	3	61	64
**Mar 07, 2022**	4	61	65
**Mar 08, 2022**	3	72	75
**Mar 09, 2022**	4	92	96
**Mar 10, 2022**	11	74	85
**Mar 11, 2022**	5	87	92
**Mar 12, 2022**	1	65	66
**Mar 13, 2022**	41	130	171
**Mar 14, 2022**	9	133	142
**Mar 15, 2022**	5	198	203
**Mar 16, 2022**	8	156	164
**Mar 17, 2022**	57	205	262
**Mar 18, 2022**	8	372	380
**Mar 19, 2022**	17	494	511
**Mar 20, 2022**	24	736	760
**Mar 21, 2022**	31	868	899
**Mar 22, 2022**	4	981	985
**Mar 23, 2022**	4	982	986
**Mar 24, 2022**	29	1587	1616
**Mar 25, 2022**	38	2233	2271
**Mar 26, 2022**	45	2633	2678
**Mar 27, 2022**	50	3454	3504
**Mar 28, 2022**	96	4382	4478
**Mar 29, 2022**	326	5658	5984
**Mar 30, 2022**	355	5299	5654
**Mar 31, 2022**	358	4145	4503
**Apr 01, 2022**	260	6051	6311
**Apr 02, 2022**	438	7789	8227
**Apr 03, 2022**	425	8585	9010
**Apr 04, 2022**	268	13088	13356
**Apr 05, 2022**	311	16767	17078
**Apr 06, 2022**	322	19661	19983
**Apr 07, 2022**	824	20401	21225
**Apr 08, 2022**	1015	22611	23626
**Apr 09, 2022**	1006	23939	24945
**Apr 10, 2022**	914	25173	26087
**Apr 11, 2022**	994	22349	23343

**Apr 12, 2022**	1189	25141	26330
**Apr 13, 2022**	2573	25147	27720
**Apr 14, 2022**	3200	19873	23073
**Apr 15, 2022**	3590	19925	23515
**Apr 16, 2022**	3238	21582	24820
**Apr 17, 2022**	2417	19831	22248
**Apr 18, 2022**	3084	17334	20418
**Apr 19, 2022**	2494	16408	18902
**Apr 20, 2022**	2634	15863	18497
**Apr 21, 2022**	1931	15698	17629
**Apr 22, 2022**	2736	20635	23371
**Apr 23, 2022**	1401	19657	21058
**Apr 24, 2022**	2472	16984	19456
**Apr 25, 2022**	1661	15319	16980
**Apr 26, 2022**	1606	11956	13562
**Apr 27, 2022**	1292	9330	10622
**Apr 28, 2022**	5487	9547	15034
**Apr 29, 2022**	1249	8932	10181
**Apr 30, 2022**	788	7086	7874

Daily new infections are the sum of symptomatic and asymptomatic cases.

Supplementary Table S4. Daily stringency index in Shanghai.

Date	C1	C2	C3	C4	C5	C6	C7	C8	Stringency index
**Mar 01, 2022**	0	0	0	0	0	0	0	75	9.38
**Mar 02, 2022**	0	0	0	0	0	0	0	75	9.38
**Mar 03, 2022**	0	0	0	0	0	0	0	75	9.38
**Mar 04, 2022**	0	0	0	0	0	0	0	75	9.38
**Mar 05, 2022**	0	0	0	0	0	0	0	75	9.38
**Mar 06, 2022**	0	0	0	0	0	0	0	75	9.38
**Mar 07, 2022**	0	0	0	0	0	0	0	75	9.38
**Mar 08, 2022**	0	0	0	0	0	0	0	75	9.38
**Mar 09, 2022**	0	0	0	0	0	0	0	75	9.38
**Mar 10, 2022**	0	0	0	0	0	0	0	75	9.38
**Mar 11, 2022**	0	0	0	0	0	0	0	75	9.38
**Mar 12, 2023**	0	0	0	0	0	0	0	75	9.38
**Mar 13, 2022**	66.67	33.33	50	50	50	33.33	0	75	44.79
**Mar 14, 2022**	66.67	33.33	50	50	50	33.33	0	75	44.79
**Mar 15, 2022**	66.67	33.33	50	50	50	33.33	0	75	44.79
**Mar 16, 2022**	66.67	33.33	50	50	50	33.33	0	75	44.79
**Mar 17, 2022**	66.67	33.33	50	50	50	33.33	0	75	44.79
**Mar 18, 2022**	66.67	33.33	50	50	50	33.33	0	75	44.79
**Mar 19, 2022**	66.67	33.33	50	50	50	33.33	0	75	44.79
**Mar 20, 2022**	66.67	33.33	50	50	50	33.33	0	75	44.79
**Mar 21, 2022**	66.67	33.33	50	50	50	33.33	0	75	44.79
**Mar 22, 2022**	66.67	33.33	50	50	50	33.33	0	75	44.79
**Mar 23, 2022**	66.67	33.33	50	50	50	33.33	0	75	44.79
**Mar 24, 2022**	66.67	33.33	50	50	50	33.33	0	75	44.79
**Mar 25, 2022**	66.67	33.33	50	50	50	33.33	0	75	44.79
**Mar 26, 2022**	66.67	33.33	50	50	50	33.33	0	75	44.79
**Mar 27, 2022**	66.67	33.33	50	50	50	33.33	0	75	44.79
**Mar 28, 2022**	83.33	50	75	62.50	75	83.33	25	75	66.15
**Mar 29, 2022**	83.33	50	75	62.50	75	83.33	25	75	66.15
**Mar 30, 2022**	83.33	50	75	62.50	75	83.33	25	75	66.15
**Mar 31, 2022**	83.33	50	75	62.50	75	83.33	25	75	66.15
**Apr 01, 2022**	100	100	100	100	100	100	100	75	96.88
**Apr 02, 2022**	100	100	100	100	100	100	100	75	96.88
**Apr 03, 2022**	100	100	100	100	100	100	100	75	96.88
**Apr 04, 2022**	100	100	100	100	100	100	100	75	96.88
**Apr 05, 2022**	100	100	100	100	100	100	100	75	96.88
**Apr 06, 2022**	100	100	100	100	100	100	100	75	96.88
**Apr 07, 2022**	100	100	100	100	100	100	100	75	96.88
**Apr 08, 2022**	100	100	100	100	100	100	100	75	96.88
**Apr 09, 2022**	100	100	100	100	100	100	100	75	96.88
**Apr 10, 2022**	100	100	100	100	100	100	100	75	96.88
**Apr 11, 2022**	100	100	100	100	100	100	100	75	96.88

**Apr 12, 2022**	100	100	100	100	100	100	100	75	96.88
**Apr 13, 2022**	100	100	100	100	100	100	100	75	96.88
**Apr 14, 2022**	100	100	100	100	100	100	100	75	96.88
**Apr 15, 2022**	100	100	100	100	100	100	100	75	96.88
**Apr 16, 2022**	100	100	100	100	100	100	100	75	96.88
**Apr 17, 2022**	100	100	100	100	100	100	100	75	96.88
**Apr 18, 2022**	100	100	100	100	100	100	100	75	96.88
**Apr 19, 2022**	100	100	100	100	100	100	100	75	96.88
**Apr 20, 2022**	100	100	100	100	100	100	100	75	96.88
**Apr 21, 2022**	100	100	100	100	100	100	100	75	96.88
**Apr 22, 2022**	100	100	100	100	100	100	100	75	96.88
**Apr 23, 2022**	100	100	100	100	100	100	100	75	96.88
**Apr 24, 2022**	100	100	100	100	100	100	100	75	96.88
**Apr 25, 2022**	100	100	100	100	100	100	100	75	96.88
**Apr 26, 2022**	100	100	100	100	100	100	100	75	96.88
**Apr 27, 2022**	100	100	100	100	100	100	100	75	96.88
**Apr 28, 2022**	100	100	100	100	100	100	100	75	96.88
**Apr 29, 2022**	100	100	100	100	100	100	100	75	96.88
**Apr 30, 2022**	100	100	100	100	100	100	100	75	96.88

Supplementary Table S5. Daily new infections in the 16 districts of Shanghai.

Date	Jingan	Huangpu	Pudong	Hongkou	Changning	Putuo	Xuhui	Yangpu
**Mar 01**	0	0	0	0	0	1	0	0
**Mar 02**	0	0	0	0	0	6	0	0
**Mar 03**	0	0	0	0	0	9	0	0
**Mar 04**	0	1	0	0	0	1	0	0
**Mar 05**	0	1	2	0	0	1	3	0
**Mar 06**	1	1	2	0	0	5	5	0
**Mar 07**	0	0	2	0	0	0	20	1
**Mar 08**	2	0	3	0	1	2	9	0
**Mar 09**	5	2	10	1	1	1	7	2
**Mar 10**	2	1	10	6	1	4	8	0
**Mar 11**	3	3	9	3	1	0	12	0
**Mar 12**	1	3	35	0	2	1	1	0
**Mar 13**	14	13	37	4	1	7	18	3
**Mar 14**	0	2	1	1	0	0	3	0
**Mar 15**	0	0	2	0	0	0	2	0
**Mar 16**	3	9	43	10	4	4	7	2
**Mar 17**	5	23	35	6	1	11	35	3
**Mar 18**	13	15	65	19	7	22	41	8
**Mar 19**	15	37	135	7	12	25	60	14
**Mar 20**	0	2	1	0	1	0	1	0
**Mar 21**	45	48	170	23	26	32	133	10
**Mar 22**	28	59	237	13	20	21	86	3
**Mar 23**	48	20	217	26	11	34	108	11
**Mar 24**	71	102	195	29	59	38	167	21
**Mar 25**	2	23	1915	4	4	9	3	11
**Mar 26**	108	159	323	49	29	69	332	24
**Mar 27**	70	56	1424	27	46	71	280	35
**Mar 28**	103	283	2502	8	115	82	92	49
**Mar 29**	180	112	2187	73	36	112	1088	98
**Mar 30**	100	363	2198	84	130	148	400	99
**Mar 31**	162	124	2404	126	258	17	226	174
**Apr 01**	189	247	2597	60	38	249	631	135
**Apr 02**	326	658	2056	344	100	389	1035	275
**Apr 03**	344	827	3669	189	104	325	498	374
**Apr 04**	60	981	6993	609	39	259	1231	225
**Apr 05**	317	663	8054	411	92	489	925	626
**Apr 06**	543	1050	8447	670	359	1035	1106	633
**Apr 07**	382	1382	9041	594	854	958	2073	601
**Apr 08**	665	2607	7286	378	614	1094	1629	701
**Apr 09**	602	548	11130	349	757	653	1150	1080
**Apr 10**	603	1761	6732	1238	405	1001	3185	1877
**Apr 11**	552	2223	8306	1375	393	1878	1771	1420

**Apr 12**	1056	1804	11049	931	1133	1170	1108	1159
**Apr 13**	220	1408	15027	1488	957	475	1492	1185
**Apr 14**	266	2013	11656	930	998	264	1342	817
**Apr 15**	433	1384	10282	1497	752	426	1689	1411
**Apr 16**	1134	1565	10791	1027	706	1331	1532	860
**Apr 17**	842	1896	7740	1166	739	1225	1553	887
**Apr 18**	1010	3323	8831	1092	680	371	572	481
**Apr 19**	1733	3084	5646	753	618	528	1608	766
**Apr 20**	1130	3097	4465	832	726	555	1447	2005
**Apr 21**	2064	1752	4655	885	496	489	1366	543
**Apr 22**	786	2843	7961	997	713	523	1275	1356
**Apr 23**	471	1959	7626	819	727	478	1722	1277
**Apr 24**	1702	1755	6181	655	749	477	1396	1235
**Apr 25**	1016	2671	3912	1270	534	418	1341	1244
**Apr 26**	1394	1228	2745	1136	419	365	904	1106
**Apr 27**	1089	1452	2993	1091	208	301	552	526
**Apr 28**	1364	2656	3472	1374	647	235	1580	808
**Apr 29**	1267	1399	2028	925	377	141	602	767
**Apr 30**	996	1156	1625	591	181	114	488	342

**Date**	**Chongming**	**Minhang**	**Jinshan**	**Baoshan**	**Songjiang**	**Qingpu**	**Jiading**	**Fengxian**
**Mar 01**	0	0	0	0	0	0	1	0
**Mar 02**	0	0	0	1	0	0	1	0
**Mar 03**	0	0	0	2	5	0	0	0
**Mar 04**	0	1	0	1	6	2	7	0
**Mar 05**	0	6	0	2	7	1	4	1
**Mar 06**	0	7	0	6	7	0	12	2
**Mar 07**	0	10	1	5	3	0	13	0
**Mar 08**	0	12	0	11	14	3	8	0
**Mar 09**	0	23	0	2	9	0	16	1
**Mar 10**	0	15	0	11	9	4	4	0
**Mar 11**	0	20	4	8	5	2	12	1
**Mar 12**	0	11	4	3	1	0	3	0
**Mar 13**	0	28	7	4	7	4	20	2
**Mar 14**	0	1	0	0	1	0	0	0
**Mar 15**	0	1	0	0	0	0	0	0
**Mar 16**	0	18	0	11	4	1	40	2
**Mar 17**	3	79	1	10	10	1	34	3
**Mar 18**	0	60	17	27	13	16	44	7
**Mar 19**	23	53	3	17	16	5	81	6
**Mar 20**	0	18	0	0	0	0	1	0
**Mar 21**	40	120	16	66	38	5	102	22
**Mar 22**	19	306	3	16	31	20	111	8
**Mar 23**	54	254	5	69	34	10	69	13

**Mar 24**	82	489	12	87	92	13	130	24
**Mar 25**	41	206	1	4	15	13	18	0
**Mar 26**	27	970	4	153	82	20	242	85
**Mar 27**	237	616	14	94	190	43	252	45
**Mar 28**	68	369	19	312	95	56	209	115
**Mar 29**	56	986	26	362	234	84	253	95
**Mar 30**	195	780	33	507	248	78	160	130
**Mar 31**	43	389	43	17	190	98	54	177
**Apr 01**	84	1029	50	46	507	87	200	162
**Apr 02**	111	822	61	480	573	224	614	158
**Apr 03**	144	937	103	463	264	220	424	121
**Apr 04**	49	1381	53	268	569	320	247	70
**Apr 05**	78	2940	82	555	801	400	491	153
**Apr 06**	63	2408	79	662	783	469	1408	267
**Apr 07**	262	2255	128	416	754	495	934	93
**Apr 08**	170	2854	90	2821	771	342	1505	97
**Apr 09**	171	4624	68	2261	500	557	369	124
**Apr 10**	55	3189	57	1839	1825	877	1405	38
**Apr 11**	57	3007	38	1024	691	331	208	68
**Apr 12**	86	4245	39	295	712	524	986	33
**Apr 13**	63	2939	34	651	657	319	721	83
**Apr 14**	60	2378	28	417	761	304	803	35
**Apr 15**	92	2037	31	1323	773	536	741	106
**Apr 16**	49	3060	21	1293	410	305	685	51
**Apr 17**	52	2402	24	1450	905	369	978	20
**Apr 18**	26	1372	7	1030	762	257	588	14
**Apr 19**	80	1602	9	957	325	389	743	60
**Apr 20**	65	1686	25	833	676	280	641	32
**Apr 21**	120	2563	30	978	493	385	776	34
**Apr 22**	117	1305	26	2033	2568	359	500	8
**Apr 23**	208	877	44	2886	773	424	751	16
**Apr 24**	95	1229	68	2506	548	251	601	7
**Apr 25**	474	717	22	1786	641	379	543	12
**Apr 26**	467	749	28	1976	287	311	434	13
**Apr 27**	199	321	41	1115	231	208	292	3
**Apr 28**	38	422	28	1243	402	269	488	6
**Apr 29**	269	516	19	1197	134	156	268	26
**Apr 30**	308	431	11	1133	105	128	259	4

Supplementary Table S6. The Omicron cases peaking date、EI and population of each district in Shanghai.

District	the date of peaking	Effective Interval (EI)	Population (*10000)
**Jingan**	Apr 21, 2022	20	97.57
**Huangpu**	Apr 20, 2022	19	66.2
**Pudong**	Apr 13, 2022	16	568.15
**Hongkou**	Apr 14, 2022	13	75.75
**Changning**	Apr 12, 2022	11	69.31
**Putuo**	Apr 11, 2022	10	123.98
**Xuhui**	Apr 11, 2022	10	111.31
**Yangpu**	Apr 11, 2022	10	124.25
**Chongming**	Apr 07, 2022	10	63.79
**Minhang**	Apr 11, 2022	10	265.35
**Jinshan**	Apr 06, 2022	9	82.28
**Baoshan**	Apr 09, 2022	8	223.52
**Songjiang**	Apr 09, 2022	8	190.97
**Qingpu**	Apr 09, 2022	8	127.14
**Jiading**	Apr 08, 2022	7	183.43
**Fengxian**	Apr 03, 2022	6	114.09

Population information is from the seventh national population census

Supplementary Table S7. Daily subway ridership of each district in Shanghai.

District	Daily subway ridership				
	Apr 18, 2015 Saturday	Apr 19, 2015 Sunday	Apr 22, 2015 Wednesday	Apr 23, 2015 Thursday	Apr 24, 2015 Friday
**Pudong**	1377457	1152531	1973473	1962125	2071716
**Xuhui**	886461	734761	1211410	1191835	1264453
**Jingan**	681097	597587	941280	942166	996443
**Huangpu**	782415	627119	893713	886328	953056
**Minhang**	663753	597381	831994	823760	893802
**Changning**	500679	412097	739678	732458	768845
**Putuo**	436150	366340	595978	593085	613605
**Baoshan**	399426	338882	520456	517868	541181
**Hongkou**	361236	300660	492646	493499	513425
**Yangpu**	323327	266286	388740	386820	415962
**Songjiang**	217269	186576	250004	246818	269363
**Jiading**	203319	177323	232934	232032	247363
**Qingpu**	56896	57773	106088	102790	110319
**Fengxian**	0	0	0	0	0
**Jinshan**	0	0	0	0	0
**Chongming**	0	0	0	0	0

Subway ridership dataset of Shanghai was shared by Chinese Software Developer Network.

Supplementary Figure S1. The change of time-dependent effective reproductive number in Shanghai's Omicron wave.

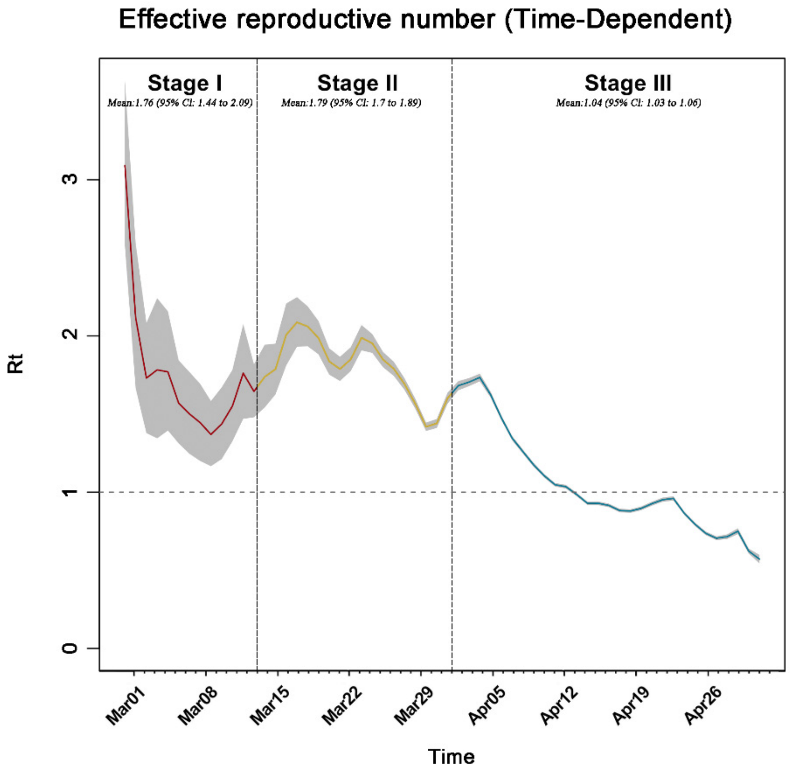

Supplementary Figure S2. Distribution of subway stations whose daily ridership on five days in April, 2015 were used for population mobility calculation in each district.

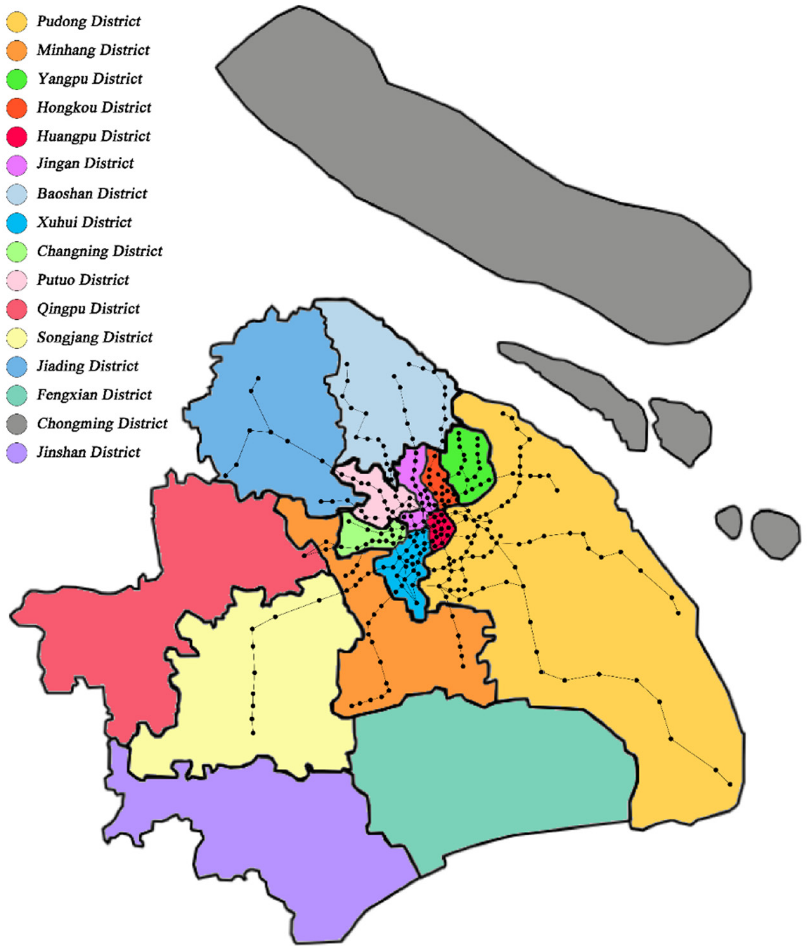

Supplement: Supplementary file 1 [file vaccines-10-01392-s001.zip › vaccines-1849991-supplementary.pdf]
